# Inhibitory effect of a gel paste containing surface pre-reacted glass-ionomer (S-PRG) filler on the cariogenicity of *Streptococcus mutans*

**DOI:** 10.1038/s41598-021-02924-6

**Published:** 2021-12-06

**Authors:** Ryota Nomura, Takahiro Kitamura, Saaya Matayoshi, Jumpei Ohata, Yuto Suehiro, Naoki Iwashita, Rena Okawa, Kazuhiko Nakano

**Affiliations:** 1 Department of Pediatric Dentistry, Osaka University Graduate School of Dentistry, Suita, Osaka Japan; 2 Department of Pharmacology, School of Veterinary Medicine, Azabu University, Sagamihara, Kanagawa Japan

**Keywords:** Microbiology, Bacteriology

## Abstract

Surface pre-reacted glass-ionomer (S-PRG) filler is a bioactive functional glass that releases six different ions. Although several dental materials containing S-PRG filler have been developed, few self-care products containing S-PRG filler have been reported. We investigated the inhibitory effects of PRG gel paste containing S-PRG filler on *Streptococcus mutans*, a major pathogen of dental caries. PRG gel paste inhibited bacterial growth of *S. mutans* in a concentration-dependent manner, and all *S. mutans* were killed in the presence of ≥ 1% PRG gel paste. Additionally, it was difficult for *S. mutans* to synthesize insoluble glucan from sucrose in the presence of 0.1% PRG gel paste. A biofilm formation model was prepared in which slices of bovine enamel were infected with *S. mutans* after treatment with or without PRG gel paste. Biofilm formation was inhibited significantly more on the enamel treated with PRG gel paste than on enamel without PRG gel paste (*P* < 0.001). The inhibitory effects on bacterial growth and biofilm formation were more prominent with PRG gel paste than with S-PRG-free gel paste, suggesting that PRG gel paste may be effective as a self-care product to prevent dental caries induced by *S. mutans*.

## Introduction

Dental caries is still the most common infectious disease in the world^1^. Recently, poor oral health has been recognized as being closely associated with quality of life^2, 3^. To prevent dental caries and maintain oral health, daily toothbrushing at home is important^4, 5^. To remove bacteria adhering to the tooth surface, mechanical removal in combination with antimicrobial toothpastes has been reported to be effective in dental caries prevention^6, 7^.

Surface pre-reacted glass-ionomer (S-PRG) filler is a bioactive functional glass that releases six ions: borate (BO_3_^3−^), aluminum (Al^3+^), silicate (SiO_3_^2-^), strontium (Sr^2+^), sodium (Na^+^) and fluoride (F^−^)^8^. S-PRG filler has been used in various dental materials including composite resins, cements, and bonding agents^9, 10^. S-PRG filler has some beneficial functions for maintaining oral health, such as anti-plaque formation, acid neutralization, and enamel remineralization^11–13^. In our previous study, ions released from S-PRG eluate, which was the supernatant after mixing S-PRG filler with sterile distilled water, could inhibit bacterial growth and biofilm formation of *Streptococcus mutans*, a major pathogen of dental caries^14^.

Development of products containing S-PRG filler has been mainly focused on dental materials, with few self-care products reported^9, 10^. Recently, a gel paste containing S-PRG filler (PRG gel paste) has been developed^15^. An experiment using enamel test pieces prepared from human extracted teeth indicated that treatment of the enamel surface with PRG gel paste may be effective for caries control via an inhibitory effect on tooth demineralization^15^. However, the effect of PRG gel paste on bacterial growth and biofilm formation induced by *S. mutans* remains unknown.

In the present study, the inhibitory effect of PRG gel paste on the major cariogenic properties of *S. mutans* was analyzed using in vitro assays to assess bacterial growth and glucan synthesis. Additionally, the effect of PRG gel paste on biofilm formation by *S. mutans* was evaluated after PRG gel paste was applied to the surface of bovine enamel test pieces.

## Results

### Effect of PRG gel paste on bacterial growth

**Figure 1:**
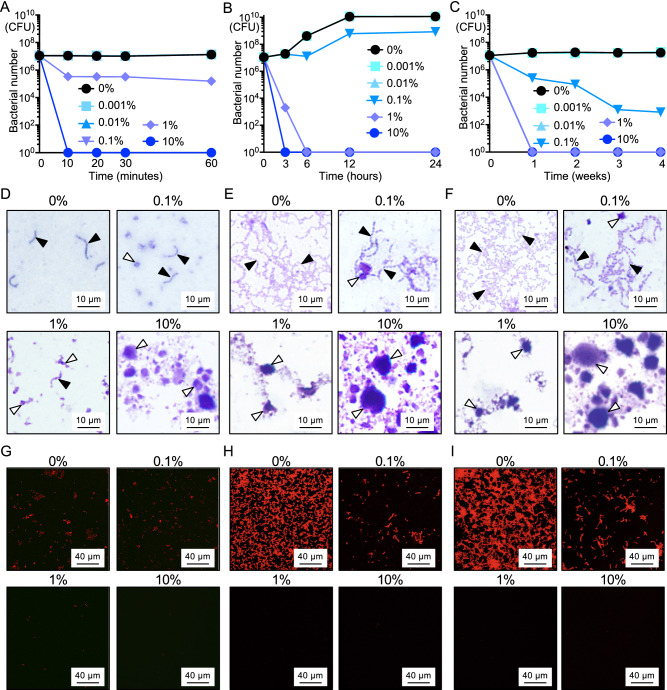
Bacterial growth of *S. mutans* in the presence of PRG gel paste. (**A**–**C**) Bacterial growth of *S. mutans* in the presence of PRG gel paste at multiple time points, including short durations (0–60 min) (**A**), medium durations (3–24 h) (**B**), and long durations (1–4 weeks) (**C**) after the start of the experiment. (**D**–**F**) Representative stereoscopic images of Giemsa-stained *S. mutans* in the presence of PRG gel paste at multiple time points, including 30 min (**D**), 12 h (**E**), and 1 week (**F**) after the start of the experiment. The concentration of PRG gel paste is shown above each image. Black and white arrowheads indicate *S. mutans* and PRG gel paste, respectively. (**G**–**I**) Representative confocal laser scanning microscopic images of *S. mutans* in the presence of PRG gel paste at multiple time points, including 30 min (**G**), 12 h (**H**), and 1 week (**I**) after the start of the experiment. The concentration of PRG gel paste is shown above each image. Bacterial cells are stained red. The confocal laser microscope images in (**G**–**I**) were taken using LSM510 (Carl Zeiss, Oberkochem, Germany) (https://www.zeiss.co.jp/corporate/home.html).

First, the inhibitory effect of PRG gel paste on the bacterial growth of *S. mutans* MT8148, which was added to brain heart infusion (BHI) broth at a concentration of 1.0 × 10^7^ CFU/ml, was chronologically examined. In the presence of 10% PRG gel paste, almost all *S. mutans* were killed in the first 10 min (Fig. 1A). The number of *S. mutans* in the presence of 1% PRG gel paste was approximately 1/30 of that in the absence of the paste after 10 min. The presence of PRG gel paste at concentrations of 0.001%–0.1% did not affect the bacterial growth after 60 min. After 6 h, almost all *S. mutans* were killed by 1% PRG gel paste, and the bacterial growth of *S. mutans* was inhibited by 0.1% PRG gel paste (Fig. 1B). In the long term, bacterial growth did not recover in the presence of 1% and 10% PRG gel paste, and the number of *S. mutans* was reduced in a time-dependent manner in the presence of 0.1% PRG gel paste (Fig. 1C). These inhibitory effects of PRG gel paste on *S. mutans* were visually confirmed by stereoscopic images of Giemsa staining (Fig. 1D–F), as well as confocal laser scanning microscopic images (Fig. 1G–I).

### Effect of PRG gel paste on *S. mutans* in the presence of sucrose

**Figure 2:**
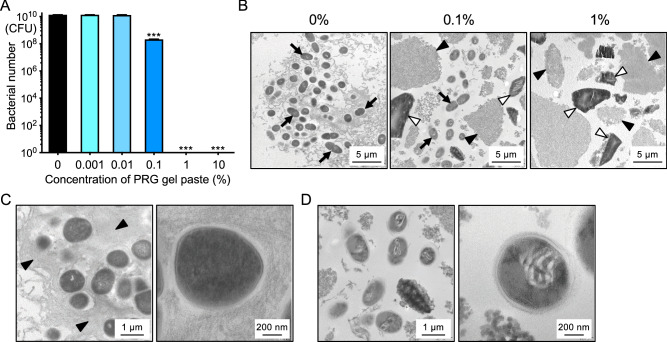
*S. mutans* and PRG gel paste in the presence of sucrose. (**A**) Bacterial growth of *S. mutans* in the presence of sucrose and PRG gel paste. Significant differences, ***P < 0.001 versus 0% PRG gel paste. (**B**) Transmission electron microscopic (TEM) images of *S. mutans* and PRG gel paste. The concentration of PRG gel paste is shown above each image. Black arrows indicate *S. mutans*, and white and black arrowheads indicate S-PRG filler and anhydrous scolic acid, respectively, both of which are contained in PRG gel paste. TEM images are shown of *S. mutans* in the presence of 0% (**C**) and 0.1% (**D**) PRG gel paste. Arrowheads indicate insoluble glucan.

An inhibitory effect on the growth of *S. mutans* was also observed in the presence of 1% sucrose after incubation for 24 h at 37 °C (Fig. 2A). Transmission electron microscopic (TEM) images revealed the presence of many *S. mutans* cells in the absence of PRG gel paste (Fig. 2B), while a small number of bacteria were found in the gaps between the gel components in the presence of 0.1% PRG gel paste. No bacteria were found in the presence of 1% PRG gel paste. Additionally, *S. mutans* cells were covered with copious quantities of glucan in the absence of PRG gel paste (Fig. 2C), while little glucan was formed around the bacteria in the presence of 0.1% PRG gel paste (Fig. 2D). Furthermore, abnormal changes in the cytoplasmic structure of *S. mutans* were observed in the presence of 0.1% PRG gel paste.

### Effect of PRG gel paste on biofilm formation using plastic plates

**Figure 3:**
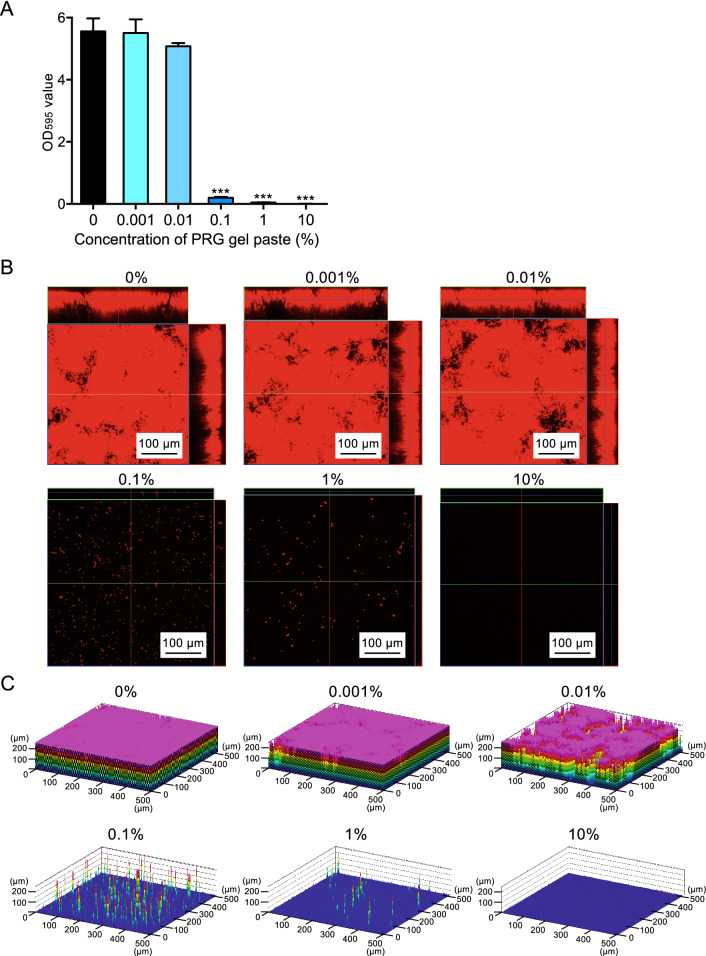
Biofilm formation of *S. mutans* in the presence of PRG gel paste. (**A**) Quantity of biofilm formation. Significant differences, ***P < 0.001 versus 0% PRG gel paste. (**B**,**C**) Representative confocal scanning laser microscopic images, in which bacterial cells are stained red (**B**), and schematics (**C**) of formed biofilms are shown. The concentration of PRG gel paste is shown above each image. The confocal laser microscope images in (**B**,**C**) were taken using LSM510 (Carl Zeiss, Oberkochem, Germany) (https://www.zeiss.co.jp/corporate/home.html).

PRG gel paste inhibited the synthesis of glucan induced by *S. mutans*, and therefore the effect of PRG gel paste on in vitro biofilm formation was analyzed. *S. mutans* at a final concentration of 1.0 × 10^7^ CFU/ml was added to plastic plates containing BHI broth and 1% sucrose with or without PRG gel paste and cultured at 37 °C for 24 h. In the presence of less than 0.01% PRG gel paste, *S. mutans* formed a similar biofilm as in the absence of the gel paste (Fig. 3A); however, in the presence of ≥ 0.1% PRG gel paste, biofilm formation was reduced. Confocal laser scanning microscopic images showed that thick biofilms were observed in the presence of 0%, 0.001%, and 0.01% PRG gel pastes (Figs. 3B,C), whereas *S. mutans* cells were sparse in the presence of 0.1% and 1% PRG gel paste, and no *S. mutans* cells were observed in the presence of 10% PRG gel pastes.

Next, the ability of the gel paste to eliminate biofilms already formed by *S. mutans* was evaluated. *S. mutans* was added to plastic plates containing BHI broth and 1% sucrose and was cultured at 37 °C for 24 h. The bacterial solution was then replaced with BHI broth containing PRG gel paste and incubated at 37 °C for 24 h. Although PRG gel paste at concentrations of more than 0.1% significantly reduced biofilm compared with the absence of PRG gel paste (*P* < 0.05) (Supplementary Fig. 1), even 10% PRG gel paste was not able to completely eliminate the biofilm.

### Effect of PRG gel paste on biofilm formation using slices of bovine enamel

**Figure 4:**
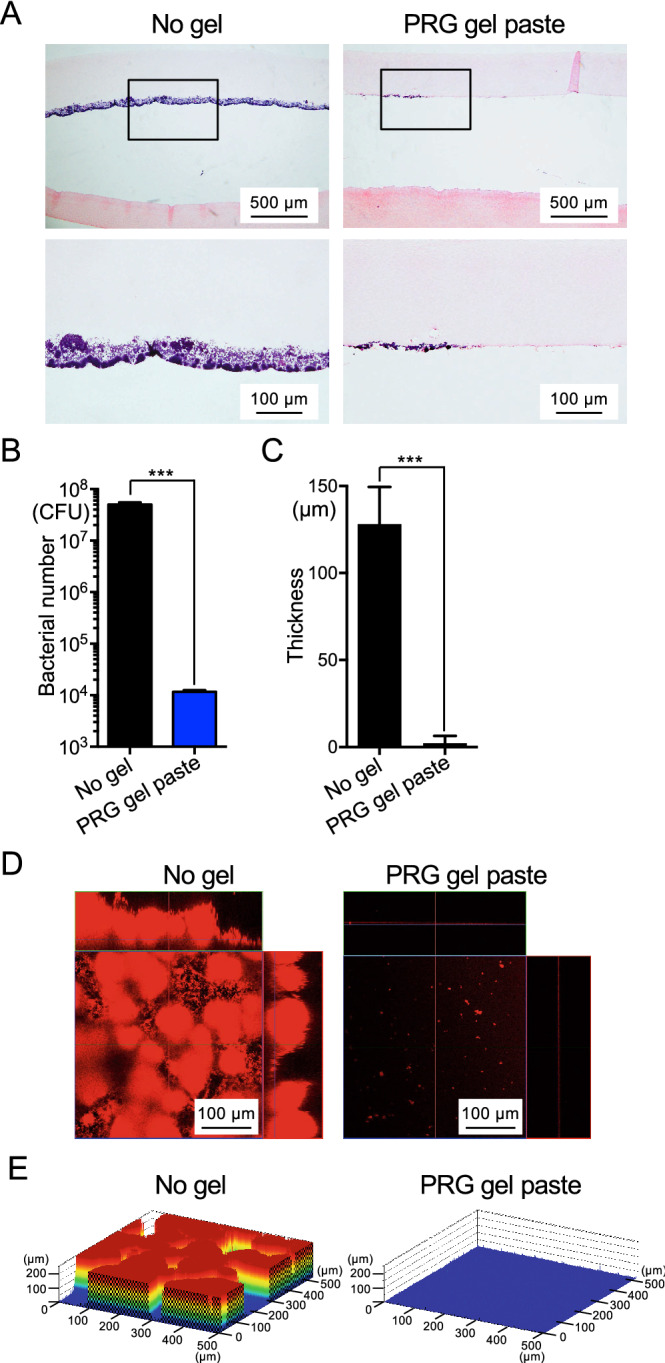
Biofilm formation of *S. mutans* on undiluted PRG gel paste-coated bovine enamel surface. (**A**) Representative histopathological images following Gram staining of sections of bovine enamel test pieces. *S. mutans* is stained purple. In the upper images, upper and lower parts of *S. mutans* indicate 3% fish collagen with 0.3% agarose and decalcified enamel, respectively, and the section below the enamel layers show dentin. The lower images show the square part of the upper images at a high magnification. (**B**) The number of *S. mutans* adhering to the bovine enamel surface. ***P < 0.001 between the groups. (**C**) Thickness of biofilm formed on the bovine enamel surfaces. ***P < 0.001 between the groups. (**D**,**E**) Representative confocal scanning laser microscopic images, in which bacterial cells are stained red (**D**), and the schematics (**E**) of bovine teeth surfaces are shown. The confocal laser microscope images in (**D**,**E**) were taken using LSM510 (Carl Zeiss, Oberkochem, Germany) (https://www.zeiss.co.jp/corporate/home.html).

To evaluate the clinical effects of PRG gel paste, slices of bovine enamel (6 mm^2^, 1 mm thick) were prepared, and the undiluted gel paste was applied to the enamel surface. The enamel test pieces were immersed in BHI containing 1% sucrose to which 1.0 × 10^7^ CFU/ml of *S. mutans* was added, and incubated at 37 °C for 24 h. Stereoscopic images of Gram staining showed formation of a uniformly thick biofilm on the untreated enamel surface (Fig. 4A), while only a partial biofilm was observed on the enamel surfaces treated with PRG gel paste. Both the number of *S. mutans* cells and the thickness of the biofilm were significantly reduced in the samples treated with PRG gel paste compared with the untreated samples (*P* < 0.001) (Fig. 4B, C). Confocal laser scanning microscopic images also showed that the thickness of the biofilm was drastically reduced by the treatment with PRG gel paste (Fig. 4D, E).

### Comparison of the effects of gel pastes with or without S-PRG filler

**Figure 5:**
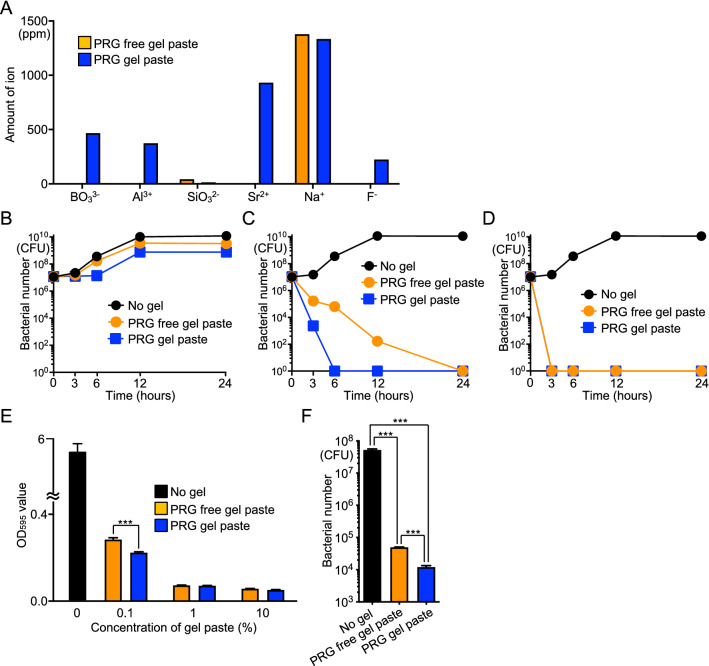
Comparison of the inhibitory effect on *S. mutans* between PRG gel paste and S-PRG filler-free gel paste. (**A**) Ion concentrations released from the gel pastes. (**B**–**D**) Bacterial growth of *S. mutans* in the presence of gel pastes of different concentrations 0.1% (**B**), 1% (**C**), and 10% (**D**). (**E**) Quantity of biofilm formation. ***P < 0.001 between the groups. (**F**) The number of *S. mutans* contained in biofilm formed on the gel paste-coated bovine teeth surface. ***P < 0.001 between the groups.

To investigate the effects of S-PRG filler in the gel paste, the inhibitory effects on *S. mutans* were compared between PRG gel paste and S-PRG filler-free gel paste. First, each gel paste was mixed with distilled water at a ratio of 1:3 for 24 h, and the amount of six ions (BO_3_^3−^, Al^3+^, SiO_3_^2−^, Sr^2+^, Na^+^ and F^−^) released into the solution was measured. The PRG gel paste solution had higher amounts of four out of six types of ions known to be released from S-PRG filler than the S-PRG-free gel paste solution (Fig. 5A). Bacterial growth in the presence of 0.1% PRG gel paste was slightly delayed compared with the growth in the presence of 0.1% S-PRG filler-free gel paste or in the absence of gel paste (Fig. 5B). Most *S. mutans* cells were killed within 6 h in the presence of 1% PRG gel paste, which required 24 h in the presence of S-PRG filler-free gel paste (Fig. 5C). In the presence of 10% gel pastes, *S. mutans* cells were killed within 3 h in the presence of the gel paste with or without S-PRG filler (Fig. 5D). The inhibitory effect in the in vitro biofilm formation assay was observed in the presence of the gel pastes regardless of the presence or absence of S-PRG filler (Fig. 5E); however, biofilm formation was significantly inhibited in the presence of 0.1% PRG gel paste when compared with the presence of 0.1% S-PRG filler-free gel paste (*P* < 0.001). Furthermore, treatment of the bovine tooth surface with the gel pastes with or without S-PRG filler resulted in significantly reduced bacterial numbers compared with no treatment (*P* < 0.001) (Fig. 5F), and the PRG gel paste had a significantly greater inhibitory effect than the S-PRG filler-free gel paste (*P* < 0.001).

## Discussion

According to the ecological plaque hypothesis, dental caries is not caused by a single pathogen, but by changes in the bacterial flora within the biofilm^16^. The biofilm on a healthy tooth surface contains mainly non-cariogenic bacteria and is not exposed to an acidic environment^17^. When sucrose is frequently consumed, the non-cariogenic bacteria try to adapt to the environmental changes, but gradually become unable to tolerate the acidic conditions. In acidic environments, either long-term or intermittent, bacteria with acid tolerance become dominant. In the present study, we focused on *S. mutans*, a major causative agent of dental caries, which can metabolize sucrose and grow in an acidic environment.

Toothpastes releasing fluoride ions are widely used for the purpose of strengthening enamel^18^, and toothpastes releasing zinc and silver ions have been developed to prevent halitosis and kill bacteria, respectively^19, 20^. Most toothpastes release F^−^ alone or another single ion in addition to F^−^; however, PRG gel paste releases six types of ions (BO_3_^3−^, Al^3+^, SiO_3_^2−^, Sr^2+^, Na^+^ and F^−^). In the present study, we analyzed the effect of PRG gel paste on the major cariogenic properties of *S. mutans*: bacterial growth and biofilm formation.

PRG gel paste inhibited the bacterial growth of *S. mutans*, and the number of bacteria decreased immediately after the start of the experiment in the presence of ≥ 1% PRG gel paste, and no viable *S. mutans* cells were observed in the presence of 10% PRG gel paste. Our results indicate that *S. mutans* on the tooth surface may be immediately eliminated when undiluted PRG gel paste is used as a prophylaxis paste. Furthermore, the growth of *S. mutans* may be inhibited when a lower concentration of PRG gel paste remains in contact with the tooth surface.

In the presence of sucrose, most *S. mutans* were killed by ≥ 1% PRG gel paste. Additionally, 0.1% PRG gel paste inhibited the synthesis of insoluble glucan by *S. mutans*, a process that is closely associated with biofilm formation^21^. In fact, strong inhibition of biofilm formation by *S. mutans* was also observed in the presence of 0.1% PRG gel paste. However, PRG gel paste was not able to completely eliminate the biofilm already formed by *S. mutans*. Therefore, it would be better to use the gel paste in combination with mechanical removal of biofilm such as toothbrushing.

Bovine teeth are widely used to assess enamel decalcification and remineralization^22–24^. However, relatively few studies have used bovine enamel to evaluate the biofilm formation of *S. mutans*^25, 26^. Biofilm formation is often studied with in vitro assays using plastic plates or glass chambers^27, 28^. In comparison with these in vitro assays, sliced bovine enamel may better reproduce the situation of polishing the tooth surface with prophylaxis paste, and may be more suitable to evaluate the effects of various toothpastes or coating materials.

We showed that treatment of bovine enamel with undiluted PRG gel paste inhibited the biofilm formation of *S. mutans*. In addition, enamel remineralization was observed when human enamel was treated with undiluted PRG gel paste^15^. On the basis of these studies, we are planning to use undiluted PRG gel paste as a prophylaxis paste. The undiluted PRG gel paste contains 5% S-PRG filler. Many dental materials such as bonding agents, cements, and composite resins contain S-PRG filler at the same or higher concentrations. These dental materials have been clinically applied, with no reports of harm to the human body. In addition, 1% of S-PRG filler was less toxic to osteoblastic cell lines than 1% of silica filler^29^. When S-PRG filler-containing sealer or silica filler-containing sealer was implanted in rat subcutaneous tissue, the accumulation of inflammatory cells was lower in the case of S-PRG filler-containing sealer than that of silica filler-containing sealer^29^. Furthermore, the safety of S-PRG filler has been demonstrated by administration experiments using the silkworm, which has similar susceptibility to chemical compounds as humans^30^.

In our previous study, a biofilm formation assay was performed using *S. mutans* on saliva-coated plates in the presence or absence of S-PRG eluate^14^, demonstrating the strong inhibitory effect of S-PRG eluate on biofilm formation. The fact that the S-PRG eluate retained this effect even in the presence of saliva components indicates that the ions released from the S-PRG filler may not be markedly neutralized by saliva. Therefore, in a clinical situation, PRG gel paste may also inhibit insoluble glucan synthesis and biofilm formation by *S. mutans* in the presence of saliva. To confirm this effect, clinical studies on human subjects should be planned in the future.

Some ions in saliva, such as calcium (Ca^2+^) and phosphate (PO_4_^3−^), are involved in acid neutralization and remineralization of teeth^31^. When multiple ions from the S-PRG filler are present in saliva, the ion saturation of the saliva increases. In general, as the ion saturation increases, the equilibrium shifts to avoid saturation^32^. Therefore, the presence of S-PRG filler may facilitate the penetration of ions from the saliva into the tooth surface. Consequently, the saturation of ions in the presence of S-PRG filler may create an environment that facilitates the uptake of calcium and phosphate ions from saliva into the teeth.

Focusing on Gram-positive bacteria, S-PRG filler decreased the expression of *S. mutans* genes involved in sugar metabolism^14^. S-PRG filler also had an inhibitory effect on *Streptococcus oralis* and *Streptococcus gordonii*^33^, and may inhibit sugar metabolism by oral streptococci. In contrast, *Porphyromonas gingivalis*, a major Gram-negative periodontopathic bacterium, is unable to metabolize sugar and uses protease activity to degrade proteins as energy sources^34, 35^. S-PRG filler can inhibit the protease activity of *P. gingivalis*^36^. Furthermore, S-PRG filler can inhibit the adhesive properties and proteinase production of *Candida albicans*, a fungus known to be involved in opportunistic infections and systemic diseases in the elderly population^12, 37^. These results suggest that the ions released from PRG gel paste may have an inhibitory effect on both Gram-positive and Gram-negative bacteria that constitute biofilms in the human oral cavity, although this effect should be investigated using larger numbers of bacterial species in the future.

In the present study, we prepared S-PRG filler-free paste, which contained the same components as the PRG gel paste, but without the S-PRG filler. The PRG gel paste showed greater inhibitory effects than the S-PRG filler-free gel paste, which confirmed the effectiveness of the S-PRG filler. However, S-PRG filler-free gel paste was also found to exert an inhibitory effect on *S. mutans*, possibly because of the presence of sodium lauryl sulfate in the gel paste. Sodium lauryl sulfate, a surfactant widely used as a foaming agent in toothpaste, has an inhibitory effect on various bacteria^38^. Some surfactants, including sodium lauryl sulfate, have been reported to cause inflammation of the skin or oral mucosa^39, 40^. Cell culture experiments revealed that less than 0.01% sodium lauryl sulfate had a toxic effect on gingival fibroblasts^41^, while 1% S-PRG filler was not toxic to fibroblasts^42^. Therefore, surfactant-free toothpastes with antimicrobial effects from the S-PRG filler rather than from the surfactant should be developed in the future.

The inhibitory effect of S-PRG filler on bacteria depends on the amount of ions released^43^, which varies depending on the material containing the S-PRG filler. Therefore, it is important to detect and specify the amount of ions released from the gel paste used in the present study. We recently developed a toothbrush containing S-PRG filler that could inhibit the adhesion of *S. mutans* and easily exfoliate *S. mutans* adhering to the toothbrush monofilaments^44^. However, the inhibitory effect beyond the toothbrush was weak because only a limited amount of ions were released from the S-PRG filler in the toothbrush. In contrast, the amount of each ion released from the PRG gel paste is much higher than that released from monofilaments, resulting in a substantial inhibitory effect on *S. mutans*. Thus, the combination of PRG gel paste and the toothbrush may inhibit *S. mutans* adhering to both the toothbrush and the tooth surface, and may be an effective tool for preventing dental caries at home.

In summary, high concentrations of PRG gel paste eliminated *S. mutans* in a short period of time. Lower concentrations of PRG gel paste reduced the number of *S. mutans* cells and the amount of insoluble glucan, which resulted in inhibition of biofilm formation. These results suggest that PRG gel paste is effective in improving oral health through its inhibitory effect against *S. mutans*.

## Methods

### PRG gel paste

PRG gel paste was provided by SHOFU Inc. (Kyoto, Japan). The main component of PRG gel paste is S-PRG filler, which is produced by reacting multifunctional glass (porous SiO_2_ glass coated fluoroboroaluminosilicate glass) with polyacrylic acid solution, with the result that various ions are existed in the stable glass ionomer phase in the glass particles^8^. Other components of the prophylaxis paste are glycerin and sorbitol solution as humectants, silicic anhydride as an abrasive compound, carboxymethyl cellulose as a binding material, and sodium lauryl sulfate as a foaming agent. The concentrations of Al^3+^, BO_3_^3−^, Na^+^, SiO_3_^2−^, and Sr^2+^ ions released from the PRG gel paste were measured using an emission spectrophotometer (ICPS-8000, Shimadzu Co., Kyoto, Japan). The concentration of F^−^ was confirmed with a F^−^ electrode (Model 9609BNWP, Orion Research Inc., Beverly, MA, USA) using an ion selective electrode meter (Model 720A, Orion Research Inc.). S-PRG filler-free gel paste was also provided by SHOFU Inc., and contained the same amounts of components as the PRG gel paste, but with no S-PRG filler.

### *S. mutans* strain and culture conditions

*S. mutans* strain MT8148 (serotype *c*)^45^ was cultured on Mitis salivarius agar plates (Difco Laboratories) containing bacitracin (0.2 U/ml; Sigma-Aldrich, St. Louis, MO, USA) and 15% (w/v) sucrose (MSB agar). A single colony was inoculated into BHI broth and cultured at 37 °C for 18 h. The bacterial broth was adjusted to a final concentration of 1.0 × 10^9^ CFU/ml for use in subsequent studies.

### Bacterial growth assay

A bacterial growth assay was performed in accordance with a previously described method, with some modifications^14^. Briefly, 1.0 × 10^7^ CFU/ml of *S. mutans* was added to BHI broth with or without 1% sucrose, and PRG gel paste or S-PRG filler-free gel paste was added to the bacterial broth to a final concentration of 0%, 0.001%, 0.01%, 0.1%, 1%, and 10%. After incubation at 37 °C for short durations (10, 20, 30 and 60 min), medium durations (3, 6, 12 and 24 h), and long durations (1, 2, 3 and 4 weeks), the bacterial suspensions were serially diluted and cultured anaerobically on MSB plates at 37 °C for 48 h. Bacterial growth was calculated by counting the number of colonies on the MSB plate. All assays were carried out three times, and mean and standard deviation values were determined.

### Giemsa staining

Giemsa staining was performed to detect or confirm the presence of bacteria, as previously described with some modifications^46^. Briefly, a 10 μL bacterial suspension was applied to the glass slide, fixed with methanol for a few minutes, and stained with Giemsa solution for 30 min. The glass slide was washed with flowing water for 3 min, and *S. mutans* and the PRG gel paste were observed using a stereomicroscope.

### Electronic microscopic observation

*S. mutans* was adjusted to a concentration of 1.0 × 10^7^ CFU/ml and cultured in 1% sucrose-containing BHI broth with or without PRG gel paste at 37 °C for 24 h. *S. mutans* cells were then collected by centrifugation and fixed in glutaraldehyde adjusted to 2% with phosphate buffered saline (PBS). The bacterial structure was observed by TEM in accordance with previously described methods^47^.

### Evaluation with a biofilm formation assay using plastic plates

The quantity of the formed biofilms was assessed as previously described, with some modifications^14, 48^. Cultured bacteria were added at a concentration of 1.0 × 10^7^ CFU/ml in BHI broth containing 1% sucrose. PRG gel paste or S-PRG filler-free gel paste was then added to the bacterial broth to final concentrations of 0%, 0.001%, 0.01%, 0.1%, 1%, and 10%. Then, 200 µl aliquots of the bacterial suspensions were added to 96-well polystyrene microtiter plates. After incubation at 37 °C for 24 h, the plates were washed three times with PBS to remove loosely bound bacteria. Biofilms were fixed with 25% formaldehyde for 10 min and stained with 1% crystal violet (Sigma-Aldrich) in sterilized water for 15 min at room temperature. Next, the plates were washed three times and the biofilms were dissolved in 95% ethanol before quantification of OD_595_ values with an enzyme-linked immunosorbent assay microplate reader (Thermo Fisher Scientific, Waltham, MA, USA). All assays were carried out five times, and mean and standard deviation values were determined.

Additionally, the ability of the gel paste to eliminate biofilms already formed by *S. mutans* was evaluated. Cultured bacteria were added at a concentration of 1.0 × 10^7^ CFU/mL to plastic plates containing BHI broth and 1% sucrose and cultured at 37 °C for 24 h. Then, the bacterial solution was replaced with BHI broth and 1% sucrose containing PRG gel paste at each concentration. After incubation at 37 °C for 24 h, the biofilm formed was evaluated using the method described above.

### Evaluation with a biofilm formation assay using slices of bovine enamel

Anterior teeth extracted from healthy cattle after euthanasia were provided by a meat supplier. We used the bovine teeth in accordance with the animal experiment regulations of Osaka University and with the permission of the Animal Experiment Committee of the Osaka University Graduate School of Dentistry. The enamel of the bovine anterior teeth was embedded in epoxy resin, and the surface of the enamel was rotationally polished. The enamel was then sliced (6 mm^2^, 1 mm thick) using a precision sectioning saw after removal of the epoxy resin. Then 0.05 g of undiluted PRG gel paste or S-PRG filler-free gel paste was applied to the enamel test piece at 1000 rpm for 1 min using a rubber cup, and the paste was washed off with PBS. *S. mutans* primary adjusted 1.0 × 10^7^ CFU/ml was cultured on enamel test pieces at 37 °C for 24 h, which were then immersed in BHI broth containing 1% sucrose. The enamel test pieces were washed three times with PBS to remove loosely bound bacteria, and then placed in PBS to exfoliate the bacteria using sonication and a sterilized pipette. The bacterial suspension was serially diluted and cultured anaerobically on an MSB plate at 37 °C for 48 h, and the number of colonies on the MSB plate was counted. All assays were carried out four times, and mean and standard deviation values were determined.

### Preparation of tissue specimens from biofilm forming on bovine enamel

The formalin fixed enamel test pieces, on which biofilm had been formed by *S. mutans*, were embedded in 3% fish collagen (FUJIFULM Wako Pure Chemical Corporation, Osaka, Japan) with 0.3% agarose (FUJIFULM Wako Pure Chemical Corporation). The samples were fixed with 10% neutral buffered formalin, decalcified with EDTA, embedded in paraffin, and stained with Gram stain.

### Observation with confocal laser scanning microscope

Bacterial growth and biofilm formation were analyzed using confocal laser scanning microscopy, as described previously^14, 49^, with some modifications. In the bacterial growth assay, 500 μL of the cultured bacteria was collected by centrifugation, resuspended in 500 μL of PBS including 2.5 µl of 10 mM hexidium iodide (Invitrogen, Carlsbad, CA, USA), and incubated in the dark for 15 min at room temperature. Then, 20 μL of the bacterial suspension applied to the cover glass was fixed with 3% paraformaldehyde (FUJIFULM Wako Pure Chemical Corporation). For the biofilm assay, the biofilm formed on a chambered cover glass system (CultureWell™, Grace Bio Labs, Bend, OR, USA) or *S. mutans* cultured on the enamel test piece was stained with 5 µl of 10 mM hexidium iodide in 1 ml of Hanks’ balanced salt solution (Lonza, Walkersville, MD, USA) for 15 min at room temperature in the dark. The plates were then washed with PBS and fixed with 3% paraformaldehyde. Bacterial growth and biofilm formation were observed by confocal scanning laser microscopy using a LSM510 (Carl Zeiss, Oberkochem, Germany) with reflected laser light at 543 nm, as well as a DMI6000 B fluorescence microscope (Leica Microsystems GmbH) and a 63 × oil immersion objective.

### Statistical analysis

Statistical analyses were conducted using GraphPad Prism 9 (GraphPad Software Inc., La Jolla, CA, USA). Comparisons between two groups were performed using a chi-square test. Intergroup differences in each analysis were determined using analysis of variance (ANOVA). Bonferroni correction was used for post hoc analysis. Results were considered to be significantly different at *P* < 0.05.

## Supplementary Information

Supplementary Information.

## Data Availability

All data generated or analysed during this study are included in this published article.
