# Supplementary material for: Inhibitory effect of a gel paste containing surface pre-reacted glass-ionomer (S-PRG) filler on the cariogenicity of Streptococcus mutans
Source: Sci Rep. 2021 Dec 6;11:23495. doi: 10.1038/s41598-021-02924-6 (PMC8648751; doi:10.1038/s41598-021-02924-6)
Supplement: Supplementary file 1 — Supplementary Information. [file 41598_2021_2924_MOESM1_ESM.pdf]

Inhibitory effect of a gel paste containing surface pre-reacted glass-ionomer (S-PRG) filler on the cariogenicity of *Streptococcus mutans*

Ryota Nomura, Takahiro Kitamura, Saaya Matayoshi, Jumpei Ohata, Yuto Suehiro, Naoki Iwashita, Rena Okawa, and Kazuhiko Nakano

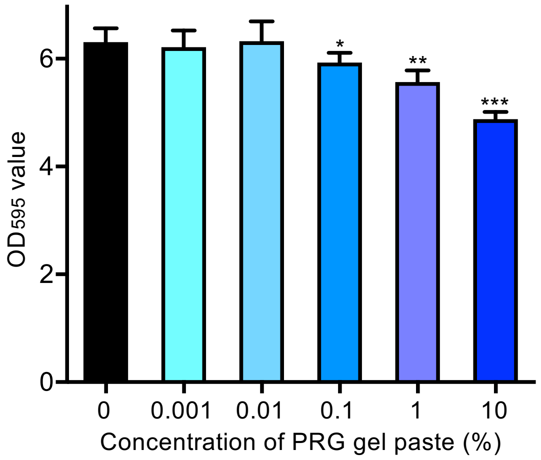

Supplementary Fig. 1 Ability of PRG gel paste to eliminate biofilms already formed by *S. mutans.* Significant differences, **P* < 0.05, ***P* < 0.01, and ****P* < 0.001 versus 0% PRG gel paste.
